# Problematic social media use and alcohol expectancies in early adolescents

**DOI:** 10.1186/s12889-023-15298-3

**Published:** 2023-03-06

**Authors:** Jason M. Nagata, Natalia Smith, Gabriel Zamora, Omar M. Sajjad, Kyle T. Ganson, Alexander Testa, Dylan B. Jackson

**Affiliations:** 1 Division of Adolescent and Young Adult Medicine, Department of Pediatrics, University of California, San Francisco, 550 16th Street, 4th Floor, Box 0503, San Francisco, CA 94143 USA; 2 Geisel School of Medicine, Dartmouth College, 1 Rope Ferry Rd, Hanover, NH 03755 USA; 3 Factor-Inwentash Faculty of Social Work, University of Toronto, 246 Bloor St W, Toronto, ON M5S 1V4 Canada; 4 Department of Management, Policy and Community Health, University of Texas Health Science Center at Houston, 7000 Fannin St, Houston, TX 77030 USA; 5 Department of Population, Family, and Reproductive Health, Johns Hopkins Bloomberg School of Public Health, Johns Hopkins University, 615 N Wolfe St, Baltimore, MD 21205 USA

**Keywords:** Social media, Adolescents, Alcohol expectancies, Problematic media use

## Abstract

**Background:**

Alcohol expectancies are beliefs regarding positive (e.g., tension reduction) or negative (e.g., loss of motor coordination) effects of alcohol. Based on Social Learning Theory, social media can influence alcohol expectancies in adolescents. In particular, problematic social media use – which can reflect elements of addiction, including mood modification, tolerance, withdrawal, conflict, and relapse – could be linked to alcohol expectancies. We aimed to determine the associations between problematic social media use and alcohol expectancies in a national (U.S.) cohort of 10-14-year-old early adolescents.

**Methods:**

We analyzed cross-sectional data from the Adolescent Brain Cognitive Development (ABCD) Study (N = 9,008) at the Year 2 assessment (2018–2020). Unadjusted and adjusted linear regression analyses were conducted to examine the associations between problematic social media use and alcohol expectancies (positive and negative), adjusting for race/ethnicity, sex, household income, parent education, sexual orientation, parental marital status, and study site. Furthermore, we computed marginal predicted probabilities to aid in interpreting findings.

**Results:**

The sample was 48.7% female and racially and ethnically diverse (43.0% non-White), with a mean age of 12.02 ± 0.66 years old. In models adjusted for confounders including both time spent on social media and problematic social media use, time spent on social media was not associated with positive or negative alcohol expectancies, but higher problematic social media use score was associated with higher positive (B = 0.045, 95% confidence interval [CI] 0.020–0.069) and negative (B = 0.072, 95% CI 0.043–0.101) alcohol expectancies scores.

**Conclusion:**

Problematic social media use was associated with both positive and negative alcohol expectancies in a demographically diverse national sample of early adolescents in the U.S. Given the small effect sizes of the current study, future studies should further examine these relationships prospectively, as well as the mechanisms linking problematic social media use to alcohol expectancies and alcohol consumption. Because alcohol expectancies are modifiable and linked with alcohol initiation, they could be a target for future prevention efforts.

**Supplementary Information:**

The online version contains supplementary material available at 10.1186/s12889-023-15298-3.

## Introduction

Excessive alcohol use accounts for over a quarter of deaths among U.S. young adults [1], and over one-third of 12th graders report past-month alcohol use [2]. Adolescents who drink alcohol before age 15 are six times more likely to develop alcohol use disorder later in life compared to those who wait until the legal drinking age of 21 [3, 4]. Alcohol expectancies are personal beliefs about behavioral, emotional, and/or cognitive effects that will occur when drinking alcohol [5]. Alcohol expectancies are learned through others and can affect behavior such as initiating or maintaining drinking [6]. Positive alcohol expectancies include beliefs about the positive effects of alcohol, while negative alcohol expectancies include beliefs about the adverse effects of alcohol [7]. Alcohol expectancies are modifiable and therefore may be important targets for efforts to prevent underage alcohol initiation among adolescents [8].

Although adolescents have access to several screen modalities, social media is uniquely positioned to influence alcohol expectancies given the public nature of alcohol content posts and the ability to “like,” react and comment on posts [9]. Social Learning Theory [10] offers a potential explanation for the effects of social media on alcohol use [11–13]. Social Learning Theory posits that learning occurs when people observe the consequences of others’ behaviors [10]. These behavioral observations can occur directly through social interactions with others or indirectly through the media. Observing peers is a major influence on adolescent health attitudes, intentions, and behaviors, particularly related to alcohol [14]. Adolescents report that 60% of their age-matched peers and 31% of their friends post alcohol content on social media [9].

Although prior studies have linked television advertisements to alcohol expectancies [5, 15], adolescents do not directly observe their own friends or classmates on television, and television is not interactive. Video games can be interactive, but the content is restricted to the game settings and generally does not focus on alcohol. Mobile phones and computers are devices that can be used for a range of functions and apps, including work, communication, videos, gaming, and social media, and therefore are a less specific exposure [15].

Social media users may post about the positive effects of alcohol, such as socialization, relaxation, or tension reduction. Alcoholic beverage companies also produce advertisements on social media exclusively portraying the positive effects of alcohol. These advertisements have been shown to increase alcohol consumption in current users and make previously non-drinking adolescents more likely to start using alcohol [16, 17]. Previous studies indicated that adolescents not only have access to official alcohol pages and related content but may be specifically targeted for alcohol advertising on social media sites such as Instagram and Twitter [18, 19]. Thus, individuals with problematic social media use may form beliefs that alcohol will produce the same positive effects as seen on social media.

Though social media content often portrays alcohol in a positive light, social media use may also contribute to negative alcohol expectancies. While users may be more inclined to post about their positive experiences with alcohol [20], others may post about the negative impacts of alcohol (e.g., vomiting, experiencing hangover symptoms, and making poor decisions). Furthermore, public health agencies and alcohol abuse prevention advertisements produce content such as videos and user polls demonstrating the negative effects of alcohol [21]. While alcohol-promoting content has been shown to produce more positive alcohol expectancies, alcohol-warning content has been shown to produce negative emotions that reduce alcohol consumption [22]. Alcohol-warning content can lead social media users to believe that alcohol has negative effects, resulting in the development of negative alcohol expectancies. Negative alcohol expectancies were originally associated with less drinking [23]; however, the predictive role of negative expectancies has become less certain as other studies demonstrated negative alcohol expectancies to be associated with problematic drinking [24, 25].

One of the few studies to examine social media and alcohol expectancies did not find a significant association between time on social media or posting frequency and positive or negative alcohol expectancies [9]. However, recent scholarship on social media and screen time has noted that measures of time (e.g., hours per day) cannot capture important qualities of social media use, such as problematic use [26]. Problematic social media use can include elements of addiction, such as mood modification, tolerance, withdrawal, conflict, and relapse [27–29]. Problematic social media use also parallels to alcohol use disorder in that both can lead to functional impairment, such as failure to meet responsibilities at school or work [28–30]. Problematic social media use may have stronger associations with alcohol expectancies than time spent on social media as it captures thoughts, feelings, and behaviors related to social media.

Various factors could account for the associations between problematic social media use and alcohol expectancies, including normalization and peer influence. Problematic social media use may normalize activities such as excessive drinking, which can increase the likelihood of forming positive alcohol expectancies. Additionally, exposure to peers’ drinking on social media may create a group identity where excessive drinking is promoted. If there is a desire to affiliate with these peers and share their group identity, adolescents may develop more positive alcohol expectancies. In a previous survey, 75% of adolescents who saw pictures of teens using alcohol noted that it made them want to engage in similar behavior [31]. Lastly, adolescents with problematic social media use may be susceptible to peers’ “likes,” comments, and other reactions (e.g., emojis) [9]. Adolescents who post alcohol-related content may receive likes and positive comments on their posts that lead to the development of more positive alcohol expectancies, potentially leading to greater levels of alcohol use and alcohol-related problems [32].

Alcohol expectancies may play a critical role in the development of alcohol use disorder, mainly by shaping individuals’ alcohol-related attitudes. Positive alcohol expectancies can increase motivation to use alcohol as adolescents may believe it will result in desirable effects such as increased social belonging. If adolescents’ drinking behavior results in positive peer feedback, this may reinforce positive alcohol expectancies and make them more likely to engage in future alcohol use. In turn, adolescents may increase their alcohol consumption to achieve previously experienced positive effects, putting them at risk for tolerance formation and alcohol dependence. More positive alcohol expectancies have been associated with alcohol misuse and alcohol use disorder [33, 34].

It is also possible that problematic social media use could make adolescents less likely to engage in social gatherings where alcohol use is present, particularly in more introverted adolescents. Previous studies have shown problematic social media use to be associated with social isolation [35]. If problematic social media use leads to the adolescent withdrawing from social settings, they may be less likely to engage in social alcohol use. This is consistent with past studies that have found extroverted individuals to be more likely to engage in binge drinking relative to introverts [36].

The current study examines the association between problematic social media use and positive and negative alcohol expectancies in a diverse, national sample of 10-14-year-old adolescents participating in the Adolescent Brain Cognitive Development (ABCD) Study. We hypothesized that problematic social media use would be associated with both positive and negative alcohol expectancies.

## Methods

We analyzed cross-sectional data from the Adolescent Brain Cognitive Development (ABCD). The ABCD Study® started following a cohort of 11,875 9-10-year-olds (mean age = 12.02 years old) from 21 research sites within the U.S. at baseline (2016–2018). Further details about the ABCD Study participants, recruitment, protocol, and measures have been previously described [37]. Data analyzed in this study (Year 2 assessment; 2018–2020; 4.0 release) were collected by adolescents’ self-report. Participants with missing data for screen time use and alcohol expectancies data were excluded (Additional File; Table S1), leaving a total sample of 9,008 adolescents. Centralized institutional review board (IRB) approval was received from the University of California, San Diego (UCSD). Study sites obtained approval from their local IRBs. Caregivers provided written informed consent, and each child provided written assent.

## Measures

### Predictors

#### Social media time

Time spent on social media was collected using adolescents’ self-reported hours and minutes on any typical weekday or weekend day they spent visiting “social media apps (e.g., Snapchat, Facebook, Twitter, Instagram, TikTok, etc.)” from the ABCD Youth Screen Time Survey. Total recreational daily social media use was calculated as the weighted sum ([weekday average x 5] + [weekend average x 2])/7. Self-reported screen use demonstrated a significant moderate positive correlation with an objective passively sensed smartphone app in the ABCD Study (r = 0.49, p < 0.001) [38].

#### Problematic social media use

The ABCD Study measured problematic social media use using the Social Media Addiction Questionnaire (SMAQ), comprised of six items modeled after the Bergen Facebook Addiction Scale [27]. Sample items included questions capturing mood modification (“I use social media apps so I can forget about my problems”), salience (“I spend a lot of time thinking about social media apps or planning my use of social media apps”), relapse (“I’ve tried to use my social media apps less but I can’t”), conflict (“I use social media apps so much that it has had a bad effect on my schoolwork or job”), and tolerance (“I feel the need to use social media apps more and more”). These scales were used among participants who reported having at least one social media account. The averages of each scale were calculated. Internal consistency (Cronbach’s alpha = 0.82) and test-retest reliability (r = 0.83) were sound [27]. The single-factor model of the SMAQ demonstrated adequate fit in a confirmatory factor analysis (χ^2^(9) = 597.23, p < 0.001, comparative fit index [CFI] = 0.943, root mean square error of approximation [RMSEA] = 0.108, 90% RMSEA CI [0.101,0.115]) [29].

### Outcomes

Alcohol expectancies were assessed via adolescents’ self-report through the validated Alcohol Expectancy Questionnaire-Adolescent, Brief (AEQ-AB) [7]. The AEQ-AB questionnaire is a 7-item instrument meant to be used by clinicians and analyzed by components (positive alcohol expectancies and negative alcohol expectancies) [7]. On a 5-point Likert scale ranging from 1 to 5, youth reported how much they agreed/disagreed with statements about the effects of alcohol. Examples include, “Alcohol makes a person feel stronger and more powerful” (positive) and “Alcohol can hurt how well a person gets along with others” (negative). See Additional File Table S2 for a full list of the items and responses. We averaged scores on particular components as recommended in the literature [7]. For the positive component, we averaged scores from items 1, 2, 4 and 6 and for the negative component we averaged scores from items 3, 5 and 7. The minimum value is 1 and the maximum is 5. The higher the score on a component or scale, the higher the expectancy.

### Covariates

Potential sociodemographic confounders in the screen time-alcohol expectancies relationship were selected based on previous literature [39, 40]. We adjusted for age (years), sex (female, male), sexual minority status (no, yes, questioning, don’t understand the question, declined to answer), race/ethnicity (White, Latino/Hispanic, Black, Asian, Native American, other), household income (greater than or less than $75,000 U.S. dollars), highest parent education (high school or less vs. college or more), parental marital status (partnered/married, unpartnered/single) and study site.

### Statistical analysis

**Fig. 1:**
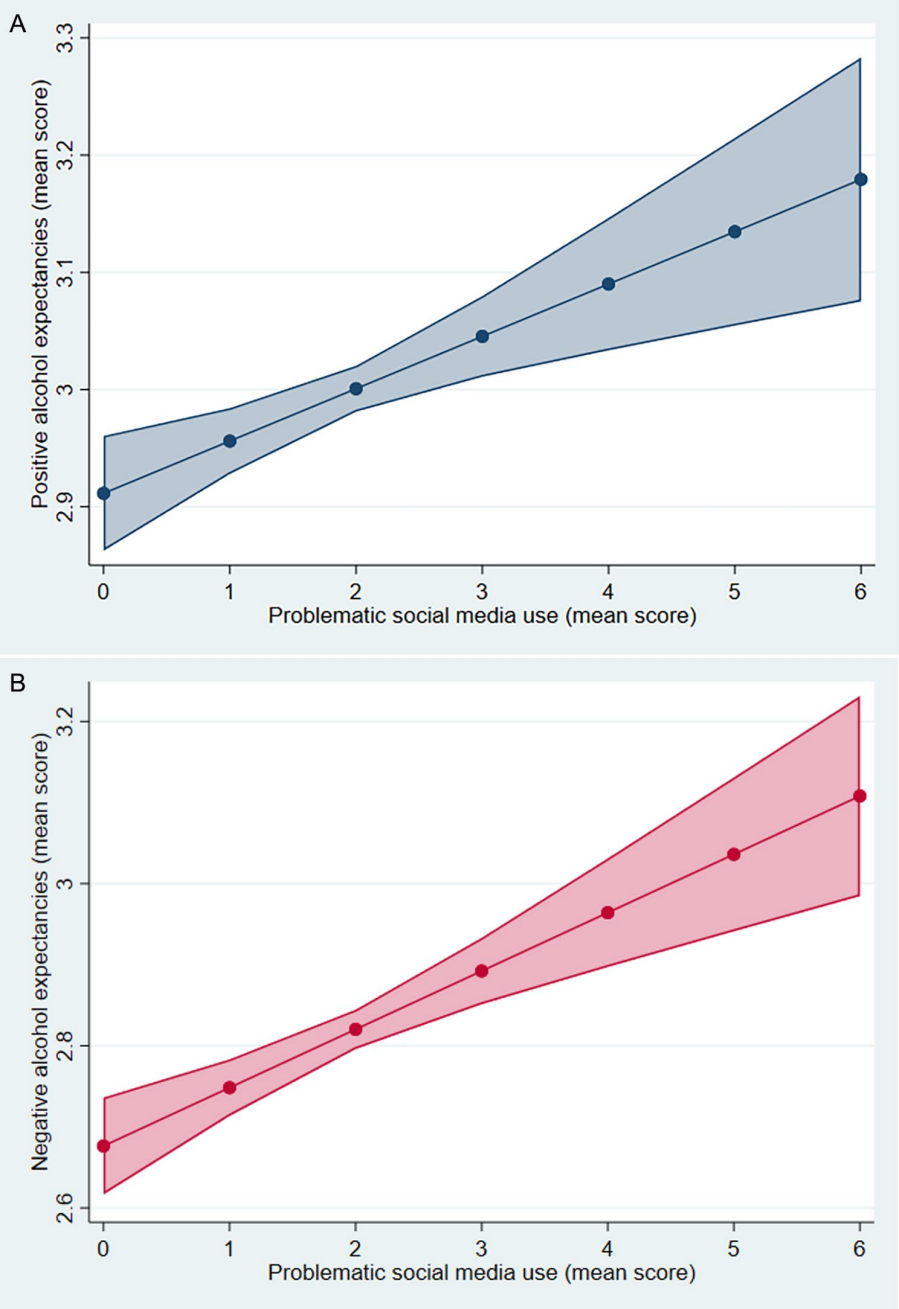
Predicted scores of problematic screen use and alcohol expectancies. (A) Problematic social media score and positive alcohol expectancies. (B) Problematic social media scores and negative alcohol expectancies

All statistical analyses were conducted in 2022 using Stata 17.0 (StataCorp). Average scores on a particular component (positive alcohol expectancies; negative alcohol expectancies) were calculated as described above [7]. Adjusted linear regression analyses were conducted to estimate cross-sectional associations between social media use variables and alcohol expectancies (positive and negative), adjusted for age, race/ethnicity, sexual orientation, household income, parent education, parent marital status, and study site [5, 24]. For each outcome, we ran three models with different exposure combinations: Model 1: social media time only; Model 2: problematic social media use only; and Model 3: social media time and problematic social media use. In Model 3, we checked for multicollinearity; however, variance inflation factors for the two exposures were < 1.20, so we retained both exposures in the model [41]. Marginal predicted probabilities were computed following the Model 3 linear regressions to get standardized estimates of alcohol expectancies among adolescents with problematic social media use to aid with interpretation of the findings (Fig. 1). Propensity weights were applied to match key sociodemographic variables in the ABCD Study to the American Community Survey from the U.S. [42]. Given the large sample size, two-sided alpha was set at p < 0.01.

## Results

Among the 9,008 adolescents of ABCD Study® that met inclusion criteria, 48.7% of the participants were female and 43.0% racial/ethnic minorities (Table 1). Average time spent on social media was 0.71 (SD = 1.62) hours per day. Mean positive and negative alcohol expectancies were 2.99 (SD = 0.61) and 2.80 (SD = 0.78), respectively.

**Table 1:** Sociodemographic, screen time, and alcohol expectancies characteristics of Adolescent Brain Cognitive Development (ABCD) Study participants (N = 9,008) at Year 2 assessment 2016–2018

Sociodemographic characteristics	Mean (SD) / %
Age (years)	12.02 (0.66)
Sex (%)	
Female	48.67
Male	51.33
Sexual minority (%)	
No	87.65
Yes	4.45
Questioning (Maybe)	3.77
Don’t understand the question	2.99
Declined to answer	1.14
Race/ethnicity (%)	
White	57.36
Latino / Hispanic	18.74
Black	14.44
Asian	5.18
Native American	3.04
Other	1.23
Household income (%)	
Less than $75,000	45.92
$75,000 and greater	54.08
Parents’ highest education (%)	
High school education or less	13.43
College education or more	86.57
Parents’ marital status (%)	
Married/partnered	71.87
Not married/unpartnered/single	28.13
**Social media**	
Social media time (hours per day)	0.71 (1.62)
Social Media Addiction Questionnaire Score^a^	1.84 (0.90)
**Alcohol Expectancies**	
Positive	2.99 (0.61)
Negative	2.80 (0.78)

ABCD propensity weights were applied based on the American Community Survey from the US Census. SD = standard deviation

Asked among a subset who reported social media use (n = 5,642)

Table 2 shows results from linear regression models examining associations between social media variables and alcohol expectancies. Time spent on social media was not associated with positive alcohol expectancies and was weakly associated with negative alcohol expectancies (Model 1). Problematic social media use score was associated with higher positive and negative alcohol expectancies scores (Model 2). In models including both time spent on social media and problematic social media use (Model 3), time spent on social media was not associated with positive or negative alcohol expectancies, but problematic higher social media use score was associated with higher positive (B = 0.045, 95% confidence interval [CI] 0.020–0.069) and negative (B = 0.072, 95% CI 0.043–0.101) alcohol expectancies scores.

**Table 2:** Associations among social media and alcohol expectancies in the Adolescent Brain Cognitive Development (ABCD) Study, 2018–2020

	Positive Expectancies		Negative Expectancies	
	B (95% CI)	p	B (95% CI)	p
**Model 1**				
Social media time (hours per day)	0.001 ( -0.010; 0.011)	0.909	**0.018 (0.005; 0.032)**	**0.007**
**Model 2**				
Social Media Addiction Questionnaire Score^a^	**0.038 (0.015; 0.061)**	**< 0.001**	**0.076 (0.048; 0.104)**	**< 0.001**
**Model 3** ^b^				
Social media time (hours per day)	-0.010 ( -0.022; 0.002)	0.106	0.005 (-0.010; 0.020)	0.504
Social Media Addiction Questionnaire Score^a^	**0.045 (0.020; 0.069)**	**< 0.001**	**0.072 (0.043; 0.101)**	**< 0.001**

Bold indicates p < 0.01. ABCD propensity weights were applied based on the American Community Survey from the US Census.

The estimated B coefficent in the cells represents abbreviated output from a series of linear regression models specifying the column header as the outcome variable and the row header as the primary predictor of interest. Thus, the table represents the output from 6 different regression models in total, adjusting for age, sex, race/ethnicity, sexual orientation, household income, parent education, parent marital status, and study site.

^a^ Asked among a subset who reported social media use (n = 5,642)

^b^ In Model 3, variance inflation factors were: social media time (1.19) and Social Media Addiction Questionnaire Score (1.17) for both positive and negative expectancies.

Predicted probabilities computed after linear regressions (Model 3) of problematic social media use and alcohol expectancies (Fig. 1) reveal that across a visual examination of the figures, alcohol expectancies scores rise along higher values of problematic social media use.

## Discussion

In a demographically diverse nationwide sample of 9,008 10-14-year-old adolescents in the United States, the present study found problematic social media use to be associated with both positive and negative alcohol expectancies, even after adjusting for potential confounders. Time spent on social media was not associated with positive or negative alcohol expectancies after considering problematic social media use.

Social Learning Theory may help explain the association between problematic screen use and positive alcohol expectancies. As posited by Social Learning Theory, adolescents learn by observing and interacting with others. Social media posts including alcohol overwhelmingly (97%) depict it in a positive social context, display people holding drinks (67%), and are mostly (54%) placed on participants’ timelines by peers rather than themselves [20]. These powerful peer comparisons and connections on social media can lead to positive alcohol expectancies.

Problematic social media use was also associated with negative alcohol expectancies. Although a majority of social media posts related to alcohol depict it in a positive social context, those depicting it in a negative context could reveal struggles with alcoholism, alcohol use disorder, or other consequences. Adolescents with problematic social media use may be sensitive to issues of addiction, tolerance, conflict, withdrawal, and relapse with their social media use. These risk behaviors could cluster together with both social media and alcohol use. Of note, negative alcohol expectancies have been shown to be associated with problematic alcohol drinking [24, 25], although findings have been mixed [23].

The differences in the associations between problematic social media use versus time spent on social media and alcohol expectancies may be explained by conceptual differences in the measures [26]. Time spent on social media does not capture specific thoughts, feelings, or behaviors related to social media, whereas the problematic social media use measure included elements relevant to addiction with parallels to alcohol use disorder such as mood modification, tolerance, withdrawal, conflict, and relapse [27, 28]. A prior study of 435 older adolescents 16–20 years in the Northeast U.S. found no association between social media time and positive alcohol expectancies [9]; our study builds upon these prior findings of no association between social media time and alcohol expectancies in a younger, larger, national sample.

The present study has several limitations. Given its cross-sectional design, causal inferences are limited. The present study adjusted for several potential confounders including race, gender, household income, parent education, sexual orientation, and parental marital status; however, residual confounders like the social media content that was consumed, as well as parenting and cultural differences, were not examined. Furthermore, underlying psychopathology or related factors (e.g., lack of self-control, attention deficit hyperactivity disorder comorbidity, social problems) could confound the relationship between problematic social media use and alcohol expectancies. There is a high chance of clustering of certain behaviors among adolescents making them more vulnerable to multiple risk behaviors [43]. The social media measures were also self-reported, which increases the risk for social-desirability bias. Given that the associations between problematic social media and alcohol expectancies were relatively small, which may be due to the young age of the cohort, further research is necessary to analyze the relationships between problematic social media and alcohol expectancies. We did not include alcohol use disorder given the young age of the current cohort, but associations between problematic social media use and alcohol use disorder could be an area of future research when the cohort is older, as prior research has shown that alcohol use disorders are most prevalent between ages 18 to 29 years [44]. The current study also had several strengths, including the large, diverse, nationwide sample that focused on early adolescents and examined a measure of problematic social media use.

The current study offers several implications within the realm of public health and healthcare. Findings are especially relevant amidst the vast increase in adolescent screen time, adolescent alcohol use, and parental permissibility of adolescent alcohol use throughout the COVID-19 pandemic [45, 46]. Our findings indicate that problematic social media use may lead to the development of positive and negative alcohol expectancies in early adolescents. Because alcohol expectancies predict alcohol initiation and are easily modifiable, they could be a prime target for prevention efforts [8]. Social media platforms could implement stricter rules regarding alcohol advertising targeted at minors, which persists despite recommendations by the Federal Trade Commission and resulting digital media alcohol advertising guidelines developed by the alcohol industry regarding age verification requirements and moderation of user-generated content [18]. Social media platforms could flag posts from minors depicting underage drinking. Parents can consider implementing parental controls on devices, enhancing privacy settings on social media, and monitoring their children’s social media activity to dissuade the propagation of underage drinking [47]. The American Academy of Pediatrics advocates for parents to develop a family media use plan, which could involve regular conversations and open communication with adolescents about online activities and promoting digital literacy [47, 48]. Digital literacy courses could provide adolescents with guidance regarding problematic social media use and the potential consequences of posting underage drinking (e.g., images discoverable by future employers, and cancel culture). Healthcare providers can ask patients and families about media use concerns, including problematic social media use as part of routine health maintenance, and encourage families to develop a family media use plan [47, 48]. Future research should examine the prospective relationships and mechanisms among problematic social media use, alcohol expectancies, alcohol use, and alcohol use disorder in later adolescence and young adulthood.

## Electronic supplementary material

Below is the link to the electronic supplementary material.

Supplementary Material 1

## Data Availability

Data used in the preparation of this article were obtained from the ABCD Study (https://abcdstudy.org), held in the NIMH Data Archive (NDA). Investigators can apply for data access through the NDA (https://nda.nih.gov/).

## Abbreviations

ABCD: Adolescent Brain Cognitive Development Study
AEQ-AB: Alcohol Expectancy Questionnaire- Adolescent, Brief
IRB: Institutional review board
MBIQ: Mobile Phone Involvement Questionnaire
SMAQ: Social Media Addiction Questionnaire
UCSD: University of California, San Diego
VGAQ: Video Game Addiction Questionnaire
