# Supplementary material for: Problematic social media use and alcohol expectancies in early adolescents
Source: BMC Public Health. 2023 Mar 6;23:430. doi: 10.1186/s12889-023-15298-3 (PMC9987130; doi:10.1186/s12889-023-15298-3)
Supplement: Supplementary file 1 — Supplementary Material 1 [file 12889_2023_15298_MOESM1_ESM.docx]

| Table S1. Comparison of participants included vs excluded | | | |
| --- | --- | --- | --- |
| Sociodemographic characteristics | Included (n = 9,008) | Excluded (n = 2,867) | p |
| Sex |  |  | 0.780 |
| Female | 48.7% | 51.3% |  |
| Male | 51.3% | 48.8% |  |
| Race/ethnicity (%) |  |  | <0.001 |
| White | 57.4% | 42.6% |  |
| Latino / Hispanic | 18.7% | 81.3% |  |
| Black | 14.4% | 85.6% |  |
| Asian | 5.2% | 94.8% |  |
| Native American | 3.0% | 97.0% |  |
| Other | 2.0% | 98.0% |  |
| Sexual minority (%) |  |  | 0.073 |
| No | 73.5% | 26.5% |  |
| Yes or questioning | 1.4% | 98.6% |  |
| Unclear | 25.1% | 74.9% |  |
| Household income (%) |  |  | <0.001 |
| $75,000 and greater | 54.1% | 45.9% |  |
| Less than $75,000 | 45.9% | 54.1% |  |
| Parents' highest education (%) |  |  | <0.001 |
| College education or more | 86.6% | 13.4% |  |
| High school education or less | 13.4% | 86.6% |  |
| Parent marital status (%) |  |  | <0.001 |
| Married/partnered | 71.9% | 28.1% |  |
| Not married/unpartnered/single | 28.1% | 71.9% |  |
| ABCD propensity weights were applied based on the American Community Survey from the US Census. | | | |

Table S2. Alcohol Expectancy Questionnaire-Adolescent, Brief (AEQ-AB)

| Items | Response Options | Expectancy |
| --- | --- | --- |
| 1. Alcohol helps a person relax, feel happy, feel less tense, and can keep a person's mind off of mistakes at school or work. | 1=Disagree Strongly; 2=Disagree Somewhat; 3=Uncertain; 4=Agree Somewhat; 5=Agree Strongly | Positive |
| 2. Alcohol can help how well a person gets along with others (makes people want to have fun together). | 1=Disagree Strongly; 2=Disagree Somewhat; 3=Uncertain; 4=Agree Somewhat; 5=Agree Strongly | Positive |
| 3. Alcohol can hurt how well a person gets along with others (makes people mean to others). | 1=Disagree Strongly; 2=Disagree Somewhat; 3=Uncertain; 4=Agree Somewhat; 5=Agree Strongly | Negative |
| 4. Alcohol helps people think better and helps coordination (people understand things better; can do things better). | 1=Disagree Strongly; 2=Disagree Somewhat; 3=Uncertain; 4=Agree Somewhat; 5=Agree Strongly | Positive |
| 5. Alcohol hurts how people think and it hurts their coordination (run into things, act silly, have a hangover). | 1=Disagree Strongly; 2=Disagree Somewhat; 3=Uncertain; 4=Agree Somewhat; 5=Agree Strongly | Negative |
| 6. Alcohol makes a person feel stronger and more powerful (easier to fight, speak in front of others, stand up to others). | 1=Disagree Strongly; 2=Disagree Somewhat; 3=Uncertain; 4=Agree Somewhat; 5=Agree Strongly | Positive |
| 7. Alcohol can make people more careless or do things that could get them into trouble (do things they feel bad about; do things they regret). | 1=Disagree Strongly; 2=Disagree Somewhat; 3=Uncertain; 4=Agree Somewhat; 5=Agree Strongly | Negative |
